# Impact of allulose on blood glucose in type 2 diabetes: A meta-analysis of clinical trials

**DOI:** 10.1016/j.metop.2024.100329

**Published:** 2024-11-07

**Authors:** Hazem Ayesh, Sajida Suhail, Suhail Ayesh

**Affiliations:** a Deaconess Health System, Evansville, IN, USA; b Gene Medical Labs, Gaza, Palestine

**Keywords:** Allulose, Type 2 diabetes, Postprandial glucose, Meta-analysis, Glycemic control

## Abstract

**Objective:**

This meta-analysis aims to evaluate the impact of allulose on blood glucose levels in patients with type 2 diabetes mellitus (T2DM). Primary outcomes include postprandial blood glucose, while secondary outcomes are time in range (TIR), time above range (TAR), fasting plasma glucose (FPG), and insulin area under the curve (AUC).

**Methods:**

A systematic search was conducted across PubMed/MEDLINE, Web of Science, Scopus, and Cochrane Library until May 20, 2024. Randomized controlled trials assessing the effect of allulose on glycemic parameters in T2DM patients were included. Data were synthesized using a random-effects meta-analysis model, and the quality of studies was assessed using the Cochrane Risk of Bias tool.

**Results:**

Six studies involving 126 participants were included. Allulose significantly reduced glucose AUC (SMD: −0.6662, 95 % CI [-1.1360, −0.1964], p = 0.0054) with moderate heterogeneity (I^2^ = 58.3 %). Insulin AUC showed a non-significant reduction (SMD: −0.3648, 95 % CI [-0.7783, 0.0488], p = 0.0839). FPG demonstrated a non-significant reduction (MD: −5.8925, 95 % CI [-20.4892, 8.7043], p = 0.4288), while TAR significantly decreased (MD: −8.8204, 95 % CI [-14.4101, −3.2307], p = 0.0020). No significant changes were observed in TIR (MD: 7.1211, 95 % CI [-1.6028, 15.8450], p = 0.1096).

**Conclusion:**

Allulose demonstrated a significant reduction in postprandial glucose levels and TAR, supporting its role as a dietary intervention for glycemic control in T2DM patients. The findings are robust, though further research is needed to confirm its long-term effects on insulin sensitivity and metabolic health.

## Highlights

• Allulose significantly reduces postprandial glucose in T2DM patients.
• Time above range (TAR) decreases with allulose, improving glycemic control.
• Allulose shows potential for improving insulin sensitivity, though more research is needed to confirm this effect.

## 1 Introduction

Type 2 diabetes mellitus (T2DM) is a chronic metabolic disorder characterized by insulin resistance and hyperglycemia, affecting millions of individuals worldwide [1]. Effective management of blood glucose levels is crucial to mitigate the long-term complications associated with T2DM, such as cardiovascular diseases, neuropathy, nephropathy, and retinopathy [2]. Traditional dietary strategies have long focused on reducing carbohydrate intake and using low-glycemic index foods to control blood sugar levels [3]. However, finding suitable alternatives that offer sweetness without triggering glycemic spikes has become a key focus in diabetes management [4]

Allulose, also known as D-psicose, is a rare sugar found naturally in small quantities in certain fruits and has a chemical structure similar to fructose [5,6]. Unlike regular sugars, allulose is not significantly metabolized by the body, thus contributing minimal calories and exerting a negligible effect on blood glucose and insulin levels [6]. Allulose can be produced through enzyme processes, the most common being the Izumoring strategy, which uses enzymes to convert regular fructose into allulose, making production more affordable and scalable. Another method uses different enzymes to build allulose from simple sugars. These enzyme-based techniques are efficient, produce fewer waste products, and are less costly than traditional methods [7].

Preliminary studies have indicated that allulose may help reduce postprandial blood glucose levels, making it a promising candidate for dietary management in individuals with T2DM [8]. Given the growing interest in low-caloric sweeteners for diabetes management, evaluating allulose's glycemic effects in clinical settings is critical.

Previous reviews have often focused on broader categories of sugar substitutes. For instance, Yuma et al. (2016) explored the effects of allulose in non-diabetic populations, showing its potential in reducing postprandial blood glucose levels in healthy individuals [9]. However, few reviews have specifically examined allulose's effects on T2DM patients, who face distinct metabolic challenges [10]. T2DM patients typically experience more pronounced insulin resistance and altered glucose metabolism, making targeted dietary strategies essential for optimizing glycemic control. Therefore, a focused evaluation of allulose in this population is necessary.

This meta-analysis aims to systematically evaluate the impact of allulose on blood glucose levels in patients with T2DM by aggregating data from multiple randomized clinical trials. The primary focus will be on postprandial blood glucose, while secondary outcomes include fasting plasma glucose (FPG), insulin area under the curve (AUC), and key glycemic metrics—Time in Range (TIR) and Time Above Range (TAR)—obtained from Continuous Glucose Monitoring (CGM) data. TIR, the percentage of time glucose levels remain within the target range (typically 70–180 mg/dL), reflects stable glycemic control with a reduced risk of complications, whereas TAR quantifies hyperglycemic exposure time, which is associated with increased risk [11]. By synthesizing available evidence, this study seeks to fill gaps in the literature and provide a robust assessment of allulose's efficacy, potentially informing dietary recommendations to enhance glycemic control in T2DM.

## 2 Methods

### 2.1 Study registration

This meta-analysis follows the PRISMA (Preferred Reporting Items for Systematic Reviews and Meta-Analyses) guidelines and was registered at https://osf.io/rtv8u [12,13].

### 2.2 Eligibility criteria

The inclusion criteria were as follows: participants diagnosed with type 2 diabetes, adults aged 18 years or older, clinical trials assessing the impact of allulose (or psicose) on blood glucose levels, and studies reporting measures of post-prandial blood glucose, TIR, TAR, FPG, and insulin AUC. The exclusion criteria included studies not reporting glucose values, observational studies, and non-human studies.

### 2.3 Search strategy

A comprehensive literature search was conducted using the following databases from inception until May 20, 2024: PubMed/MEDLINE, Web of Science, Scopus, and Cochrane Library. A detailed search strategy was developed to capture all relevant studies. The search string used was: ("Allulose" OR "psicose") AND ("type 2 diabetes") AND ("postprandial blood glucose" OR "TIR" OR "TAR" OR "FPG" OR "insulin AUC") AND ("clinical trials" OR "randomized controlled trials" OR "RCTs"). A pilot search was conducted to validate the comprehensiveness of the strategy, ensuring that seminal studies in the field were captured.

### 2.4 Study selection and screening

Two reviewers, HS and SS, independently conducted the search and screening process. The selection process consisted of three stages [1]: Title Screening: Initial screening of titles using predefined keywords [2]. Abstract Screening: Abstracts were reviewed using both computer-assisted tools and manual assessment [3]. Full-text Assessment: Full texts of the remaining articles were assessed. Any discrepancies between HS and SS were resolved through discussion, or with the help of a third reviewer, SA.

### 2.5 Data extraction and preparation

Data extraction was conducted by HS and SS using a standardized data extraction form. The following data were collected: study characteristics (publication year, country, study design, sample size, duration of follow-up), participant characteristics, intervention details, outcome measures (post-prandial blood glucose, TIR, TAR, FPG, insulin AUC), and risk of bias indicators (randomization process, blinding, incomplete outcome data, selective reporting). For studies reporting medians and interquartile ranges (IQR) or ranges, means and standard deviations (SD) were estimated using the formulas provided by Wan et al. [14]. Specifically, the mean was approximated from the median, and the SD was calculated from the confidence intervals (CI) using the formula SD = SE × √n, where SE is derived from the CI width using SE = CI width/(2 × 1.96) [15].

### 2.6 Quality assessment

The quality of included studies was assessed using the Cochrane Risk of Bias (RoB) tool [16]. This tool evaluates several domains, including random sequence generation (selection bias), allocation concealment (selection bias), blinding of participants and personnel (performance bias), blinding of outcome assessment (detection bias), incomplete outcome data (attrition bias), selective reporting (reporting bias), and other potential sources of bias. Each study was classified as having a low, unclear, or high risk of bias in each domain. Disagreements during the assessment process were resolved through discussion or by consulting a third reviewer. The overall risk of bias for each study was considered when interpreting the findings of the meta-analysis.

### 2.7 Data analysis and reporting

Data were synthesized using a random-effects meta-analysis model to account for heterogeneity between studies. Effect sizes for each outcome measure were calculated using appropriate statistical methods. Subgroup analyses and publication bias evaluations were conducted only if a sufficient number of studies were available. All statistical analyses were conducted using the R programming language (version 4.3), Meta package (version 7.0.0), Dmetar package (version 0.1.0), Robvis package (version 0.3.0), and Rstudio [[17], [18], [19], [20]]. Results were reported in accordance with the PRISMA guidelines, providing a comprehensive overview of the findings, including detailed tables and forest plots for effect sizes, heterogeneity measures, and publication bias assessments.

## 3 Results

### 3.1 Baseline characteristics of included studies

**Fig. 1:**
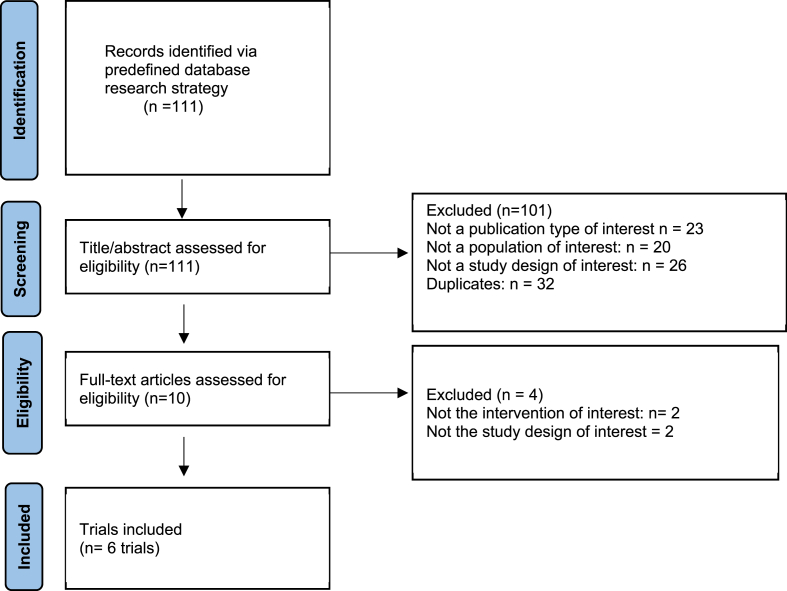
Figure 1: PRISMA flowchart for study selection

The six included studies, conducted across Thailand, Canada, Japan, South Korea, and Malaysia, enrolled a total of 126 participants [5,[21], [22], [23], [24], [25]] (see Fig. 1). Due to the use of a cross-over design and historical controls in some studies, the number of observations exceeds the number of unique participants in the subsequent analyses. The mean age of participants ranged from 55.0 to 66.0 years. The studies reported mean weights between 65.3 kg and 82.7 kg, and BMIs ranged from 24.9 to 32.2 kg/m^2^. The participants' HbA1c levels varied from 6.6 % to 9.2 %, and fasting plasma glucose levels were between 104.6 mg/dL and 148 mg/dL. The duration of interventions spanned from 5 days to 12 weeks. Different doses of allulose were examined, ranging from 5 g to 8.5 g per meal, administered either as dietary supplements or added to meals (see Table 1).

**Table 1:** Baseline characteristics and study details.

Study	Design	Country	Intervention	Study Duration	N	Gender (Male %)	Age (years)	Weight (kg)	BMI (kg/m^2^)	HbA1c (%)	Fasting Plasma Glucose (mg/dL)
**Preechasuk, 2022**	Double-blind, randomized, controlled, crossover clinical trial	Thailand	Participants consumed allulose (7 g) or aspartame twice daily for 12 weeks with a 2-week washout period between treatments.	12 weeks	16	37.5 %	54.2 (10.4)	73.3 (15.0)	28.3 (5.5)	6.6 (0.6)	110 (14)
**Noronha, 2018**	Double-blind, randomized, controlled, acute feeding equivalence design with washout periods	Canada	Participants received fructose or allulose (0 g, 5 g, or 10 g) added to a 75-g glucose solution, with blood glucose and insulin measurements.	7 weeks	22	50 %	66 (1.2)	76.2 (3.7)	27.0 (0.9)	6.7 (0.1)	124 (3.6) (converted)
**Fukunaga, 2023**	Prospective, randomized, single-blind, crossover comparative study	Japan	Participants were given a diabetic diet with and without D-allulose (8.5 g per meal) in a crossover design with washout periods between interventions.	5 days	20	70 %	61.0 (11.9)	65.6 (14.2)	25.6 (4.2)	9.2 (1.8)	148 (39) (converted)
**Tak, 2023**	Single-arm, historical-control pilot clinical trial	South Korea	Participants consumed 2 packs of diabetes-specific Oral Nutritional Supplement (ONS) including allulose (200 kcal/200 mL) for 8 weeks instead of breakfast.	8 weeks	26	53.8 %	58.23 (6.19)	67.20 (8.29)	25.59 (1.82)	–	104.6 (13.4)
**Japar, 2022**	Pilot, prospective intervention study	Malaysia	Participants consumed 8.5 g of D-allulose before iftar for 5 days during Ramadan, with continuous glucose monitoring.	10 days	12	50 %	55.2 (6.83)	82.7 (19.8)	32.2 (7.6)	6.7 (0.41)	–
**Hayashi, 2010**	Double-blind, randomized, controlled, crossover clinical trial	Japan	Participants consumed 5 g of D-psicose three times a day for 12 weeks, with blood glucose levels measured at multiple time points.	12 weeks	30	50 %	55.0 (11.4)	65.3 (13.1)	24.9 (4.4)	–	104.6 (13.4)

∗Values are presented as mean (SD) for continuous variables.

Abbreviations.

• BMI: Body Mass Index.

• HbA1c: Glycated Hemoglobin.

• N: Number of Participants.

### 3.2 Glucose and insulin AUCs

**Fig. 2:**
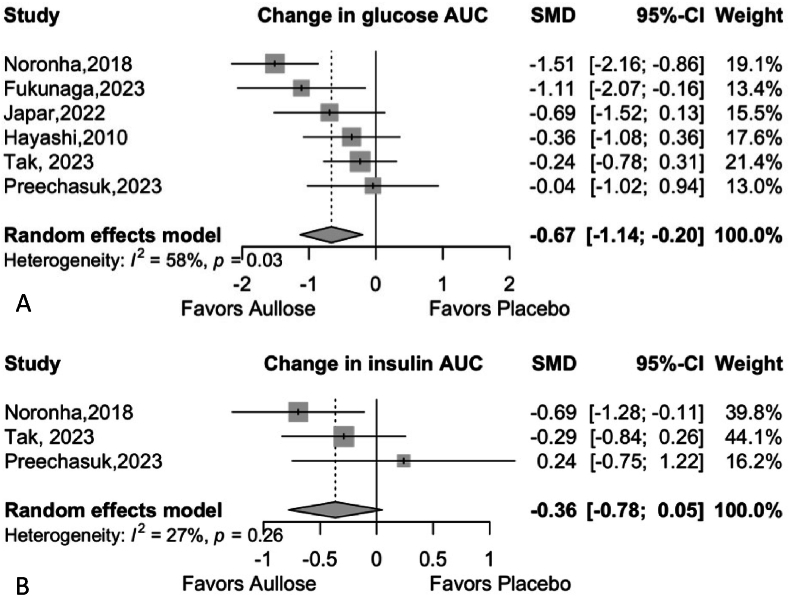
Meta-analysis of the Effect of Allulose on Glucose and Insulin Area Under the Curve (AUC). **Panel A:** This forest plot displays the change in glucose AUC. **Panel B:** This forest plot displays the change in insulin AUC. Abbreviations: AUC: Area Under the Curve, SMD: Standardized Mean Difference, CI: Confidence Interval.

Six studies were included in the analysis of glucose AUC, comprising a total of 190 observations (95 in the experimental group and 95 in the control group). The random-effects model demonstrated a significant reduction in glucose AUC, with a standardized mean difference (SMD) of −0.6662 (95 % CI [−1.1360, −0.1964], p = 0.0054). Moderate heterogeneity was detected across the studies (I^2^ = 58.3 %, τ^2^ = 0.1912, p = 0.0349), indicating some variability in effect sizes. The use of Hedges' g provided a bias-corrected SMD, and the restricted maximum-likelihood estimator was applied to estimate τ^2^ (Fig. 2). This suggests that allulose may contribute to a clinically meaningful reduction in glucose AUC, though further studies are needed to confirm the consistency of the effect.

Three studies were included in the analysis of insulin AUC, with a total of 116 observations (58 in the experimental group and 58 in the control group). The random-effects model suggested a non-significant reduction in insulin AUC, with an SMD of −0.3648 (95 % CI [−0.7783, 0.0488], p = 0.0839). Heterogeneity was low (I^2^ = 26.7 %, τ^2^ = 0.0232, p = 0.2555), indicating minimal variability between studies (Fig. 2). Although the effect on insulin AUC was not statistically significant, the trend toward reduction suggests that allulose may have a beneficial role in modulating insulin levels.

### 3.3 Glucose control parameters

**Fig. 3:**
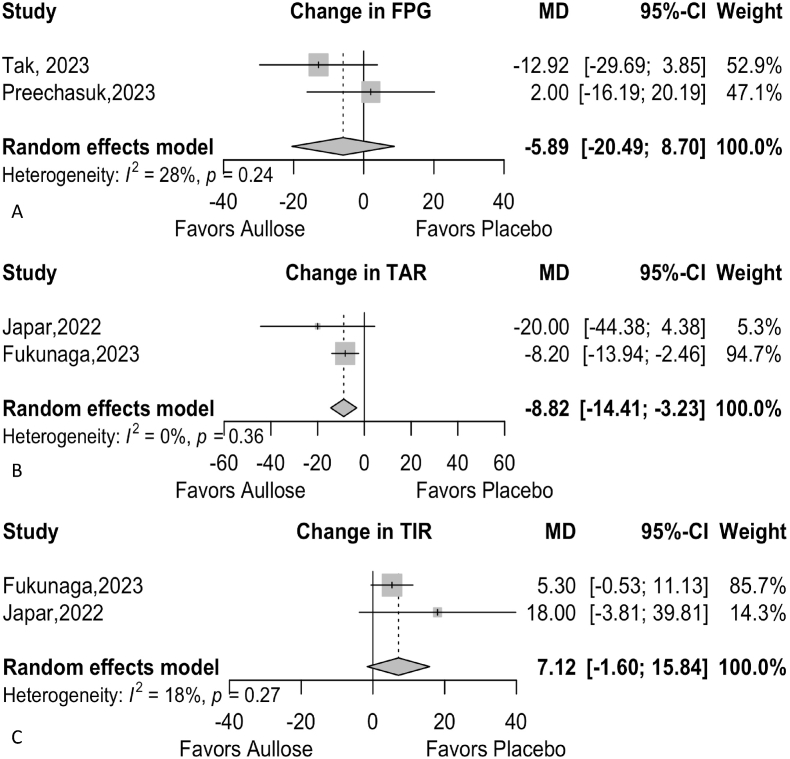
**Meta-analysis of the Effect of Allulose on Various Glycemic Parameters. Panel A:** This forest plot displays the change in fasting plasma glucose (FPG). **Panel B:** This forest plot displays the change in time above range (TAR). **Panel C:** This forest plot displays the change in time in range (TIR). Abbreviations: AUC: Area Under the Curve, SMD: Standardized Mean Difference, CI: Confidence Interval, FPG: Fasting Plasma Glucose, MD: Mean Difference, TAR: Time Above Range, TIR: Time in Range.

Two studies were included in the analysis of FPG, with a total of 68 observations. The random-effects model indicated a non-significant reduction in FPG, with an overall mean difference (MD) of −5.8925 (95 % CI [−20.4892, 8.7043], p = 0.4288). Heterogeneity was low (I^2^ = 28.4 %, τ^2^ = 31.6308, p = 0.2372) (Fig. 3). The wide confidence intervals reflect uncertainty about the effect, likely due to small sample sizes and short study durations.

In the analysis of time above range (TAR), two studies were included, with a total of 44 observations. The random-effects model revealed a significant reduction in TAR, with an overall MD of −8.8204 (95 % CI [−14.4101, −3.2307], p = 0.0020). No heterogeneity was detected (I^2^ = 0.0 %, τ^2^ = 0, p = 0.3558), indicating consistent findings across the studies (Fig. 3). This suggests that allulose may effectively reduce TAR, a key indicator of improved glycemic control.

Two studies were also included in the analysis of time in range (TIR), with 44 observations. The random-effects model showed a non-significant increase in TIR, with an MD of 7.1211 (95 % CI [−1.6028, 15.8450], p = 0.1096). Heterogeneity was low (I^2^ = 17.7 %, τ^2^ = 14.2792, p = 0.2703), suggesting that variability between studies was minimal (Fig. 3). Although the increase in TIR was not statistically significant, the trend toward improvement is encouraging and warrants further investigation.

### 3.4 Validation of statistical analysis

To ensure the robustness of our findings, we conducted a simulation-based power analysis for each outcome measure using the Metafor(4.6.0)package in R [26]. This analysis provided high power estimates for the primary and secondary outcomes, with glucose AUC at 94.6 %, insulin AUC at 91.1 %, fasting plasma glucose (FPG) at 88.3 %, time above range (TAR) at 100 %, and time in range (TIR) at 76.3 %. While most outcomes demonstrated strong power, the lower power for TIR (76.3 %) highlights a potential limitation in detecting statistically significant effects for this metric, suggesting caution in interpreting non-significant TIR findings.

To control for the risk of Type I error in multiple comparisons, we applied a Bonferroni correction to the significant outcomes for glucose AUC and TAR. After correction, the adjusted p-values were 0.0108 for glucose AUC and 0.0040 for TAR, both of which remain below the conventional significance threshold of 0.05. This correction validates the robustness of our significant findings and reinforces the clinical relevance of allulose's effects on these key glycemic parameters.

### 3.5 Summary of risk of bias assessment and sensitivity analysis

Most of the included studies demonstrated a low risk of bias across key domains, including deviations from intended interventions, missing outcome data, outcome measurement, and selective reporting. However, certain studies raised specific concerns. In particular, Japar 2022 and Tak 2023 showed some concerns in the randomization domain, which could introduce variability in baseline characteristics. Additionally, Tak 2023 and Hayashi 2010 exhibited concerns in reporting bias, with Tak 2023 having a high risk of bias due to selective reporting. These identified biases could potentially affect the reliability of the results, especially for studies with higher weight in the analysis. Nonetheless, sensitivity analyses indicate that the overall effect sizes remain consistent, suggesting that these biases have a minimal impact on the robustness of our conclusions. Given that only six studies were included in this meta-analysis, a funnel plot for publication bias and meta-regression were not performed, as these analyses typically require a larger number of studies to yield reliable results. Despite this, the analysis remains robust, supported by a leave-one-out sensitivity analysis, which indicated that the overall effect size remained stable across studies.

**Fig. 4:**
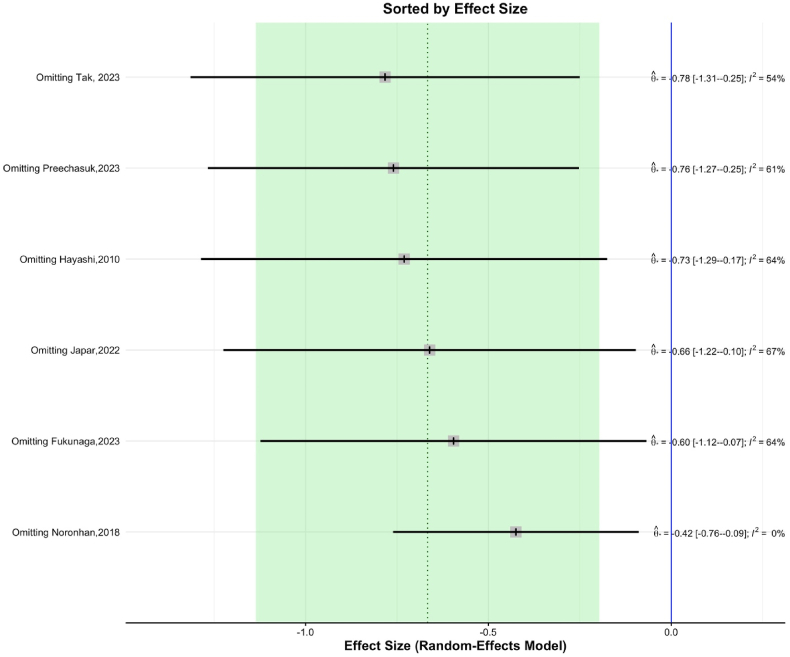
This graph shows how the omission of each study affects the overall effect size in a meta-analysis. Each line represents the effect size and 95 % confidence interval (CI) after omitting the study named on the left. The squares represent the point estimates, while the horizontal lines show the CI. The green shaded area represents the range of effect sizes. The vertical dotted line indicates the overall effect size with all studies included, and the blue line represents no effect. The I^2^ statistic indicates heterogeneity among the remaining studies when each study is omitted. (For interpretation of the references to colour in this figure legend, the reader is referred to the Web version of this article.)

**Fig. 5:**
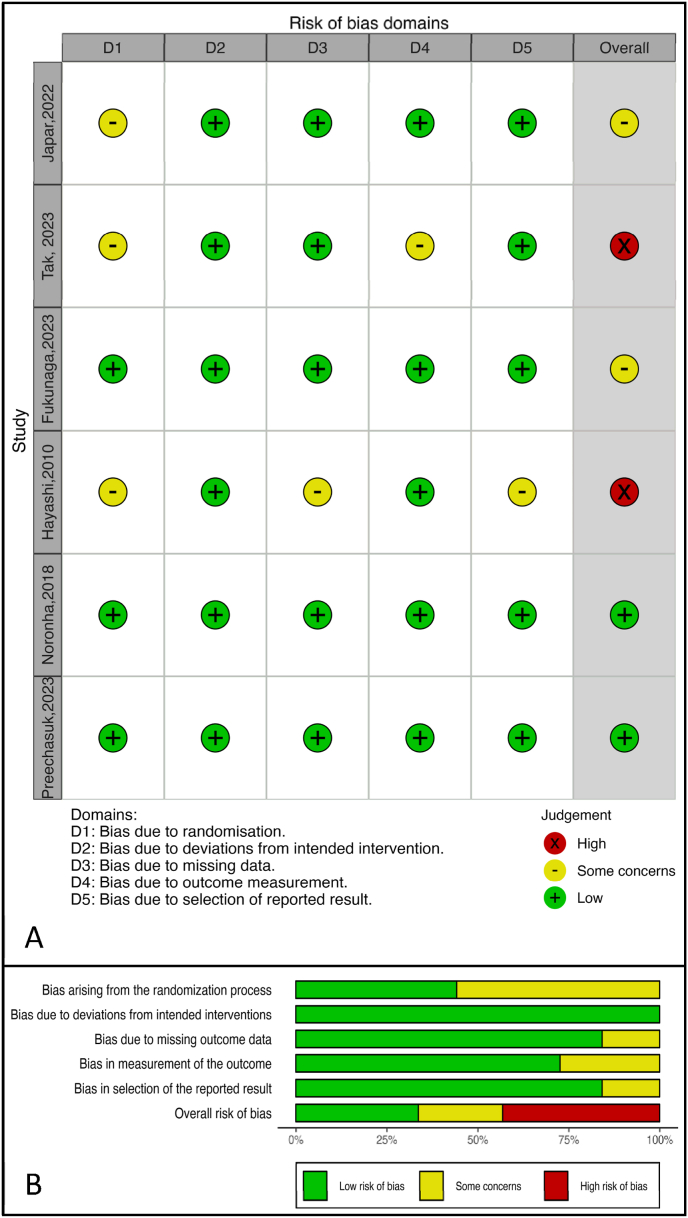
**Risk of Bias Assessment for Included Studies. Panel A**: Shows risk of bias domains for individual studies with symbols for high risk (red X), some concerns (yellow -), and low risk (green +). **Panel B**: Summarizes the proportion of studies with low risk (green), some concerns (yellow), and high risk (red) for each bias category. **Abbreviations**: **D1**: Bias due to randomization, **D2**: Bias due to deviations from intended intervention, **D3**: Bias due to missing data, **D4**: Bias due to outcome measurement, **D5**: Bias due to selection of reported result. (For interpretation of the references to colour in this figure legend, the reader is referred to the Web version of this article.)

Omitting Noronha, 2018 resulted in an SMD of −0.425 (95 % CI [−0.761, −0.089], I^2^ = 0.0 %), suggesting minimal heterogeneity. In contrast, omitting Tak, 2023 led to a larger effect size, with an SMD of −0.783 (95 % CI [−1.315, −0.251], I^2^ = 54.4 %), indicating moderate heterogeneity. This suggests that while the overall model is stable, certain studies contribute more to the observed heterogeneity (Fig. 4). Other studies showed consistent results, further reinforcing the robustness of the model (see Fig. 5).

## 4 Discussion

The results of this meta-analysis provide a comprehensive assessment of allulose's impact on various glycemic parameters in individuals with T2DM. The significant reduction in AUC observed across six studies suggests that allulose effectively lowers postprandial glucose levels, which is crucial for managing T2DM and preventing complications. The observed reduction in glucose AUC is statistically significant which makes clinical relevance of this effect is noteworthy. Allulose's ability to reduce glucose levels without significantly increasing insulin secretion may help preserve pancreatic β-cell function and improve insulin sensitivity over time, making it particularly beneficial for individuals with insulin resistance or those looking to preserve pancreatic function.

The mechanisms through which allulose exerts its effects likely involve reducing glucose absorption in the intestine by inhibiting glucose transporters and enhancing hepatic glucokinase activity, which promotes glycogen synthesis [7,8]. These mechanisms explain the reduction in glucose AUC and suggest broader metabolic benefits, potentially improving overall insulin sensitivity and glycemic control. Additionally, allulose's ability to stabilize blood glucose levels throughout the day, as suggested by the reductions in TAR and the trend towards increased TIR, points to its role in maintaining better glycemic control over time.

The lack of significant findings for fasting plasma glucose (FPG) and insulin AUC, despite observed trends, suggests that allulose's primary influence is on postprandial rather than fasting glucose. This aligns with the distinct regulatory mechanisms for insulin during fasting and postprandial states. Fasting glucose is maintained by basal insulin and hepatic glucose production, processes less sensitive to short-term dietary changes like allulose intake. Conversely, postprandial glucose is driven by rapid absorption and insulin-mediated clearance, where allulose may exhibit its greatest impact. Mechanistically, allulose appears to modulate glucose through several pathways. It likely inhibits intestinal glucose absorption via glucose transporter interactions, reducing post-meal glucose spikes. It may also stimulate GLP-1 release, which enhances insulin sensitivity and decreases glucagon levels, promoting stable glucose without significant insulin secretion [27]. Additionally, allulose may increase glucokinase activity in liver cells, encouraging glycogen storage over glucose release, thus improving postprandial control. Together, these mechanisms highlight allulose's targeted effects on postprandial glucose regulation. Prolonged studies are recommended to further assess incremental effects on fasting glucose and insulin levels.

While specific dosage recommendations for allulose are still being established, our findings suggest that individuals with T2DM may benefit from allulose as an adjunct dietary intervention to stabilize postprandial glucose levels. Future studies should focus on establishing optimal dosing across varied patient demographics to enhance clinical applicability. Although each included study is randomized, we acknowledge that factors like dietary habits and physical activity levels could act as confounders, and controlling for these in future studies could refine our understanding of allulose's effects. The manuscript now notes that while allulose naturally occurs in some fruits, human safety data remains limited; no adverse effects were reported in studies included here, yet more research across different populations is necessary to ensure its safe application. Additionally, we emphasize that clinicians should assess each patient's individual needs and monitor blood glucose closely when incorporating allulose to provide tailored, safe care. Clinicians should consider each patient's unique health profile when recommending allulose, with careful monitoring of blood glucose changes to ensure safe and effective use. Individualized dosing and observation can help optimize benefits and mitigate any unforeseen responses.

When comparing allulose with other sweeteners like sucralose and stevia, key differences emerge. Sucralose, while effective in maintaining glucose levels in healthy individuals, has been shown to increase insulin AUC in people with obesity, potentially affecting insulin sensitivity [28]. In contrast, allulose reduces glucose AUC without significantly affecting insulin AUC, making it a more favorable option for managing postprandial glucose without overstimulation of insulin secretion [23]. Stevia reduces both glucose and insulin AUC [29], but the available data suggest that allulose exhibits a trend towards lowering insulin AUC, though the effect was not statistically significant. This trend indicates that allulose may help reduce insulin secretion without overstimulating the pancreas, which could support better long-term metabolic control in individuals with T2DM. However, more studies are needed to confirm these findings regarding insulin AUC for allulose.

From a broader clinical perspective, allulose's effects can complement other common interventions for T2DM, such as dietary modifications, physical activity, and pharmaceutical treatments. While medications like metformin or glucagon-like peptide-1 (GLP-1) receptor agonists are effective in reducing postprandial glucose levels, allulose offers an additional dietary approach that may further enhance glycemic control. In particular, allulose could potentially work well alongside GLP-1 receptor agonists, given their complementary mechanisms of action. GLP-1 receptor agonists improve glycemic control by increasing insulin secretion and slowing gastric emptying, while allulose reduces glucose absorption and minimizes postprandial spikes. Together, these interventions may provide a more comprehensive approach to managing postprandial glucose levels without placing excessive demand on insulin secretion. However, more research is needed to fully understand the long-term effects of using allulose in combination with GLP-1 receptor agonists, particularly regarding insulin sensitivity and overall metabolic health.

Several limitations must be considered when interpreting these results. The small sample sizes and short duration of many included studies may limit the generalizability of the findings. Additionally, variability in study design, doses of allulose, and forms of administration likely contributed to the heterogeneity observed in some outcomes. The limited number of studies on insulin AUC for allulose further restricts the ability to draw strong conclusions in this area. Future studies should focus on assessing the long-term impacts of allulose on insulin sensitivity and glycemic control in diverse populations.

Looking ahead, future research should aim to conduct larger, long-term studies to confirm the sustained effects of allulose on glycemic control and to explore its impact on other metabolic parameters, such as lipid profiles and body weight. Standardizing the doses and forms of allulose used in clinical trials will enhance comparability and provide clearer guidelines for clinical practice. Additionally, investigating the potential effects of allulose on the gut microbiota and insulin sensitivity will offer deeper insights into its role in overall metabolic health.

In conclusion, this meta-analysis supports the efficacy of allulose in reducing postprandial glucose levels in individuals with T2DM, highlighting its potential as a valuable dietary intervention. By addressing the current limitations through larger, long-term studies, we can further understand its full potential and integrate allulose into clinical guidelines for the management of T2DM.

## CRediT authorship contribution statement

**Hazem Ayesh:** Writing – review & editing, Writing – original draft, Visualization, Validation, Supervision, Software, Resources, Project administration, Methodology, Investigation, Formal analysis, Data curation, Conceptualization. **Sajida Suhail:** Writing – review & editing, Software, Formal analysis, Data curation. **Suhail Ayesh:** Writing – review & editing, Writing – original draft, Validation, Conceptualization.

## Ethics approval

Analyses were performed on data extracted from published papers. Patient consent for publication was not required.

## Availability of data and materials

All relevant data from this study are provided within the article and supplementary materials. For any additional information, please contact the corresponding author.

## Declaration of generative AI and AI-assisted technologies in the writing process

During the preparation of this work the authors used ChatGPT in order to correct grammar. After using this tool/service, the autho(s reviewed and edited the content as needed and takes full responsibility for the content of the publication.

## Funding

No external funding was received for this publication.

## Declaration of competing interests

We declare no conflict of interest.

## Figures and Tables

**Table:** 

**T2DM**	Type 2 diabetes mellitus
**AUC**	Area under the curve
**TIR**	Time in range
**TAR**	Time above range
**FPG**	Fasting plasma glucose
**SD**	Standard deviation
**CI**	Confidence interval
**SE**	Standard error
**PRISMA**	Preferred Reporting Items for Systematic Reviews and Meta-Analyses
**RCT**	Randomized controlled trial
**RoB**	Risk of Bias
**SMD**	Standardized mean difference
**BMI**	Body mass index
**HbA1c**	Glycated hemoglobin
**GLP-1**	Glucagon-like peptide-1

## References

[1] American Diabetes A. (2022). Standards of medical care in diabetes—2022. Diabetes Care.

[2] National Institute of D (2021). Digestive, kidney D. Diabetes Overview.

[3] Bergenstal R.M., Johnson M., Powers M.A., Wynne A., Vlajnic A., Hollander P. (2008). Adjust to target in type 2 diabetes: comparison of a simple algorithm with carbohydrate counting for adjustment of mealtime insulin glulisine. Diabetes Care.

[4] Mejia E., Pearlman M. (2019). Natural alternative sweeteners and diabetes management. Curr Diabetes Rep.

[5] Hayashi N., Iida T., Yamada T., Okuma K., Takehara I., Yamamoto T. (2010). Study on the postprandial blood glucose suppression effect of D-psicose in borderline diabetes and the safety of long-term ingestion by normal human subjects. Biosci Biotechnol Biochem.

[6] Jiang S., Xiao W., Zhu X., Yang P., Zheng Z., Lu S. (2020). Review on D-allulose: in vivo metabolism, catalytic mechanism, engineering strain construction, bio-production technology. Front Bioeng Biotechnol.

[7] Chen Z., Gao X.D., Li Z. (2022). Recent advances regarding the physiological functions and biosynthesis of D-allulose. Front Microbiol.

[8] Hossain A., Yamaguchi F., Matsuo T., Tsukamoto I., Toyoda Y., Ogawa M. (2015). Rare sugar D-allulose: potential role and therapeutic monitoring in maintaining obesity and type 2 diabetes mellitus. Pharmacol Ther.

[9] Yuma T., Tokuda M., Nishimoto N., Yokoi H., Izumori K. (2023). Allulose for the attenuation of postprandial blood glucose levels in healthy humans: a systematic review and meta-analysis. PLoS One.

[10] Pang M.D., Goossens G.H., Blaak E.E. (2020). The impact of artificial sweeteners on body weight control and glucose homeostasis. Front Nutr.

[11] Wright E.E., Morgan K., Fu D.K., Wilkins N., Guffey W.J. (2020). Time in range: how to measure it, how to report it, and its practical application in clinical decision-making. Clin Diabetes.

[12] Ayesh H. (2024).

[13] Page M.J., McKenzie J.E., Bossuyt P.M., Boutron I., Hoffmann T.C., Mulrow C.D. (2021). The PRISMA 2020 statement: an updated guideline for reporting systematic reviews. BMJ.

[14] Wan X., Wang W., Liu J., Tong T. (2014). Estimating the sample mean and standard deviation from the sample size, median, range and/or interquartile range. BMC Med Res Methodol.

[15] Higgins J.P.T., Thomas J., Chandler J., Cumpston M., Li T., Page M.J. (2022/02/01).

[16] Sterne J.A.C., Savović J., Page M.J., Elbers R.G., Blencowe N.S., Boutron I. (2019). RoB 2: a revised tool for assessing risk of bias in randomised trials. BMJ.

[17] team P. RStudio (2023).

[18] Balduzzi S., Rücker G., Schwarzer G. (2019). How to perform a meta-analysis with R: a practical tutorial. Evid Base Ment Health.

[19] Harrer M, Cuijpers P, Furukawa T, Ebert DD. Dmetar: companion R package for the guide 'doing meta-analysis in R'. R package version 0102019.

[20] McGuinness L.A. (2019).

[21] Fukunaga K., Yoshimura T., Imachi H., Kobayashi T., Saheki T., Sato S. (2023). A pilot study on the efficacy of a diabetic diet containing the rare sugar D-allulose in patients with type 2 diabetes mellitus: a prospective, randomized, single-blind, crossover study. Nutrients.

[22] Japar S., Fukunaga K., Kobayashi T., Imachi H., Sato S., Saheki T. (2022). A pilot study on the effect of D-allulose on postprandial glucose levels in patients with type 2 diabetes mellitus during Ramadan fasting. Diabetol Metab Syndrome.

[23] Noronha J.C., Braunstein C.R., Glenn A.J., Khan T.A., Viguiliouk E., Noseworthy R. (2018). The effect of small doses of fructose and allulose on postprandial glucose metabolism in type 2 diabetes: a double-blind, randomized, controlled, acute feeding, equivalence trial. Diabetes Obes Metabol.

[24] Preechasuk L., Luksameejaroenchai C., Tangjittipokin W., Kunavisarut T. (2023). Short-term effects of allulose consumption on glucose homeostasis, metabolic parameters, incretin levels, and inflammatory markers in patients with type 2 diabetes: a double-blind, randomized, controlled crossover clinical trial. Eur J Nutr.

[25] Tak J., Bok M., Rho H., Park J.H., Lim Y., Chon S. (2023). Effect of diabetes-specific oral nutritional supplements with allulose on weight and glycemic profiles in overweight or obese type 2 diabetic patients. Nutr Res Prac.

[26] Viechtbauer W. (2010). Conducting meta-analyses in R with the metafor package. J Stat Software.

[27] Franchi F., Yaranov D.M., Rollini F., Rivas A., Rivas Rios J., Been L. (2021). Effects of D-allulose on glucose tolerance and insulin response to a standard oral sucrose load: results of a prospective, randomized, crossover study. BMJ Open Diabetes Research & Care.

[28] Pepino M.Y., Tiemann C.D., Patterson B.W., Wice B.M., Klein S. (2013). Sucralose affects glycemic and hormonal responses to an oral glucose load. Diabetes Care.

[29] Anton S.D., Martin C.K., Han H., Coulon S., Cefalu W.T., Geiselman P. (2010). Effects of stevia, aspartame, and sucrose on food intake, satiety, and postprandial glucose and insulin levels. Appetite.

